# Electronic medical record systems data use in decision-making and associated factors among health managers at public primary health facilities, Dodoma region: a cross-sectional analytical study

**DOI:** 10.3389/fdgth.2023.1259268

**Published:** 2024-02-12

**Authors:** Eusebi Cornelius Kessy, Stephen Mathew Kibusi, Julius Edward Ntwenya

**Affiliations:** School of Nursing and Public Health, The University of Dodoma, Dodoma, Tanzania

**Keywords:** electronic medical records, data use, decision-making, health managers, public primary health facilities, cross-sectional analytical study

## Abstract

**Background:**

Tanzania has shown some improvements in the adoption of electronic medical record (EMR) systems in public health facilities; however, the rate of utilization of data generated from EMRs among health managers is not well documented. This study aims to assess the use of electronic medical record systems data in decision-making among health managers at public primary health facilities in Dodoma Region, Central Tanzania.

**Methods:**

A facility-based quantitative cross-sectional analytical study was conducted among 308 randomly selected health managers. A self-administered questionnaire supplemented with documentary review was used. Descriptive summary statistics and bivariable and multivariable logistic regression analyses (crude and adjusted odds ratios) were used. A *P*-value of <0.05 was used to declare statistically significant associations.

**Results:**

Overall, more than a third (40.6%) of the health managers, that is 174 of the 308 included in the study, reported using data generated by EMR systems in decision-making. One-third (33.4%) of the health managers were adequately using data generated by EMR systems, of which 39.3% used data to support continuous quality improvement initiatives. Among the facilities visited, only nine (30%) had good documented EMR systems data use. Access to computers [adjusted odds ratio (AOR) = 4.72, 95% confidence interval (CI): 1.65, 13.48, *p*-value (*p*) = 0.004] and discussions on EMRs during meetings (AOR = 2.77, 95% CI: 1.01, 7.58, *p* = 0.047) were independent predictors of EMR system data use. Those who reported having EMR systems in all working areas were seven times more likely to use EMR system data (AOR = 7.23, 95% CI: 3.15, 16.59, *p* = 0.001). The respondents with good perceived EMR system information quality were more likely to use EMR system data (AOR = 2.84, 95% CI: 1.50, 5.39, *p* = 0.001) than those with poor perception. Furthermore, health managers who had excellent knowledge of computers and data use had higher odds of using EMR system data (AOR = 1.84, 95% CI: 3.38, 10.13, *p* = 0.001) compared with their counterparts.

**Conclusions:**

The findings of this study indicate that utilization of EMR system data in decision-making among health managers was optimal. It was found that training in itself is insufficient to improve use of EMR, which points to more organizational aspects of work routine as a challenge. Hence, a comprehensive approach that addresses these factors is essential for maximizing EMR system data use in decision-making.

## 1. Introduction

Electronic medical record (EMR) system, which is an integral part of the larger health management information system (HMIS), is a software that records health-related information of patients on computers. It is a program that collects, manages, and generates data that are used by authorized workers within health facilities. EMR systems provide substantial benefits to health facilities and organizations in patient management and are quickly replacing paper-based systems. They solve many of the limitations of paper-based systems and have proved to be cost-effective in improving the quality of health services (1). They improve the accessibility of health records as well as the quality and accuracy of patient information. Given these benefits, institutions have widely adopted EMR systems in healthcare globally (2).

Previously, all tasks related to the administration and management of patients, such as human resources, procurement, and clinical management, were managed using paper-based record-keeping systems, which proved to be inefficient in terms of information retrieval, security, and data quality and did not allow concurrent data access. However, paper-based systems are still used by health facilities largely because many facilities have not installed EMR systems and the health workers are more familiar with paper-based records due to the long-term use and the fact that they do not require a high level of technical knowledge and skills compared with the electronic systems (1).

An EMR system is a powerful tool for improving clinical and administrative/managerial decision-making by providing access to accurate and relevant information (3). It provides a comprehensive way to manage patients and streamline administrative tasks, and is one of the best healthcare analytics solutions as it collects and analyzes patient data to identify trends and patterns and develop predictive models (4). The increasing integration of highly diversified technologies in the healthcare field has resulted in the need for such an organized gathering of accurate data for informed decision-making in the health sector (5).

Globally there is an increasing volume of health-related data being generated, accelerating the trend towarddigitalization in health. However, there are persistent data gaps and fragmented approaches to the governance of health data in different contexts, which has contributed to inadequate data use (6). EMR systems have begun to be widely used in healthcare facilities worldwide as a data collection and aggregation tool (7). A systematic review of publications from 15 Sub-Saharan African countries revealed that about 91% reported the use of open-source healthcare software. However, the use of EMR systems in low-income countries remains in its initial stages (8, 9).

In developed countries like the USA, 94% of the hospitals use electronic health records (EHR) data in decision-making, such as for quality improvement (82%), monitoring patient safety (81%), and measuring organizational performance (77%). EHR data were least commonly used to develop approaches to query for patient data (51%), assess adherence to clinical practice guidelines (59%), and identify care gaps for specific patient populations (60%). While EHRs are used by multiple care providers and health organizations, hospital characteristics (public, private, rural, and urban) significantly impact the use of EMR data. However, there was substantial variation in the use of EHR data (10).

In Sub-Saharan Africa, a study conducted in a Malawian hospital revealed that utilization of EMR functionalities varied among departments as well as among users. Health facility workers used half of the system functions, and the most commonly used were capturing demographics (82.9%) and capturing and assessing clinical data (68.8%). These functions were frequently used because they applied to almost all the patients who visited the hospital. Gender, age, and previous computer use did not influence EMR systems usage. However, education and employment levels were predictors of EMR systems use (1).

In Tanzania, public health facilities use different EMR systems; some of the EMR systems used include the government of Tanzania hospital management information system (GoTHOMIS), Jeeva, Afya Care, and CTC2 database (11, 12). An EMR system is intended to improve and strengthen electronic data capture at the point of care to improve patient clinical care and facility management. The use of electronic data systems has led to improvements in the quality of health services delivery, including improvement in revenue collection, human resource management, supply chain management, health information management, and improved planning and decision-making at different levels of the health system (13, 14).

A study conducted in Tanzania on paper-based HMIS data use in decision-making revealed that about 56.9% of the facilities had functional HMIS, 18% of the facilities had used their data for planning and services improvement, 26.3% had disseminated data, and about 9.1% of the facilities had proper medical records. The level of the facility was associated with the use of data, with hospitals and health centers showing higher use (15). Another study (16) on paper-based data use in decision-making revealed that 60% of the respondents reported using HMIS data in decision-making, of which data was most used to compare service coverage (53%).

The government of Tanzania, through its fifth Health Sector Strategic Plan (HSSP V), has set the priority to improve the application of digital health technologies. The aim is to facilitate the attainment of high-quality health services to all citizens. Despite this, very few health facilities have installed and made use of EMR systems as data gathering and aggregation tools. Expansion of the system to all government-owned facilities is a priority (14). Owing to the limited resources, most electronic systems are still being used side by side with paper documentation, which is creating a burden on the healthcare workers (2, 17).

HSSP V prioritizes the application of digital health technologies, including the use of EMR systems in public health facilities (14). The government, through support from implementing partners, started the development and implementation of comprehensive electronic medical record systems in 2015 (18). As of now, the AfyA Care and GoT-HoMIS have been approved for scale-up country wide (19). Currently, the GoT-HoMIS system has been deployed in 1,424 healthcare facilities (20). Despite EMR systems supporting data synthesis and visualizations, the use of EMR systems in decision-making in Tanzania is still inadequate (13, 21).

The increasing adoption of the EMR system in Tanzania as a data collection and aggregation tool should be implemented together with improvements in the capacity of health managers to harness its potential. The weakness in general managerial capacity of the health system has been cited as one of the contributory factors in failing to scale up effective health interventions. Hence, understanding how health facilities are currently using data generated by EMR systems in decision-making is important as one of the policy initiatives (14). A study (16) on paper-based systems revealed that 60% of health facility workers and 38.5% of district officials use routine health information for decision-making, with the majority using it in comparing service coverage and monitoring disease trends over time and health promotion activities.

In 2023, the government introduced a data use toolkit for the health sector that insists that the availability of timely, retrievable, and accessible quality data is a cornerstone of all health systems. The health facilities are advised to conduct regular data review meetings (weekly or monthly) to understand their data, identify challenges, and set action items to improve health services. The health facility management team (HMT) has a role in conducting data use review meetings, monitoring the use of data, ensuring evidence-based planning and documentation of data use activities, and increasing the capacity of health workers in data use skills. One of the data sources insisted on in this guideline is EMR systems such as GoT-HoMIS (22).

Despite large investments to support the adoption of EMR systems in Tanzania, so far limited studies have been conducted to give an insight into the acceptance and use of data generated by EMR systems in decision-making in the study area. Therefore, this study aims to fill the knowledge gap related to EMR data utilization in decision-making and its associated factors among health facility managers in the public primary health facilities in Dodoma Region, Central Tanzania.

### 1.1. Conceptual framework

In this study, the Performance of Routine Information System Management Series (PRISM) framework was conceptualized and adapted in establishing the association between the individual, organizational, behavioral, and technical factors related with EMR systems and the utilization of the systems data in decision-making. The PRISM framework was modified to include individual factors that can influence data use in decision-making, such as age, sex, work experience, level of education, and managerial experience.

**Figure 1:**
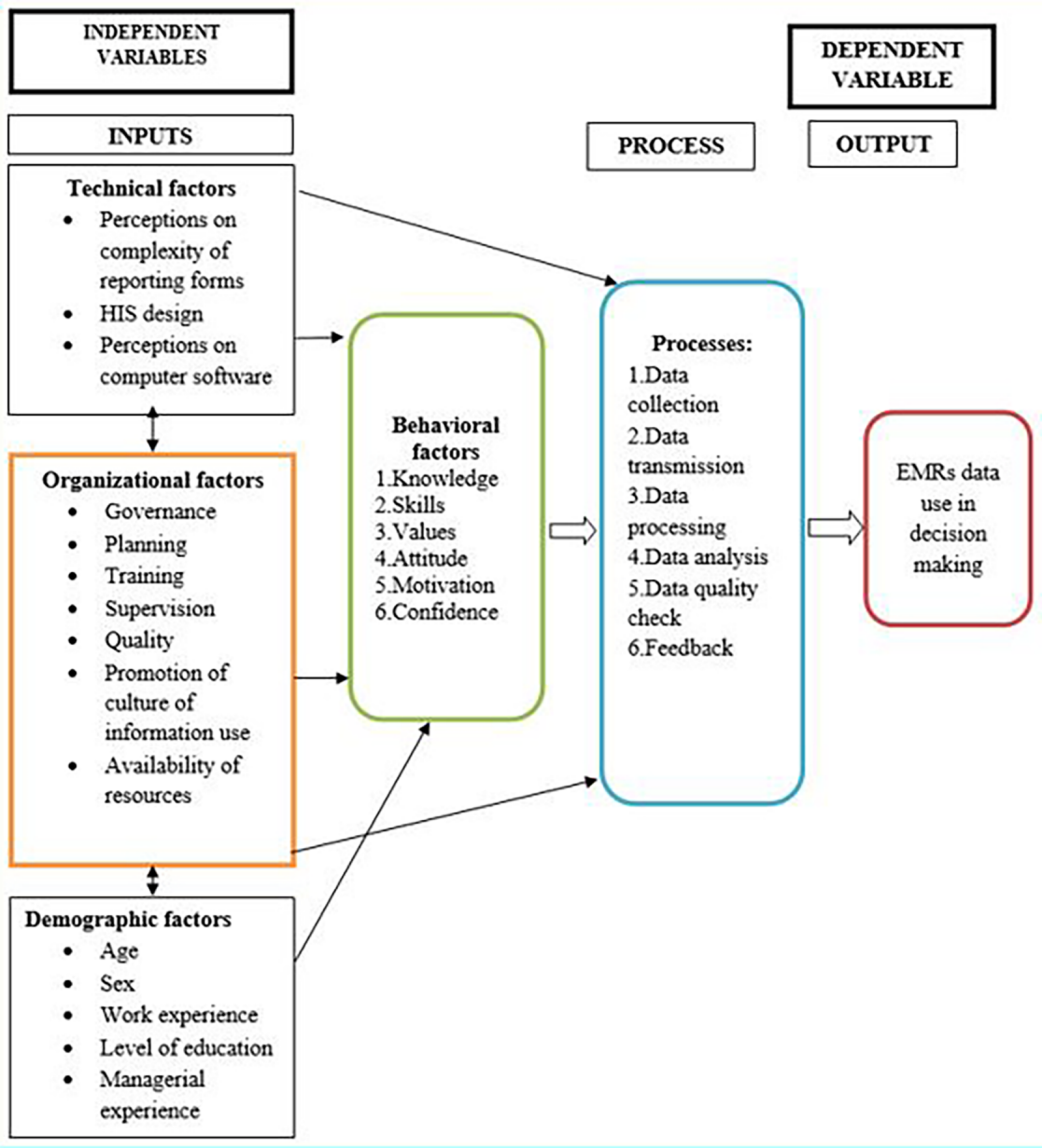
Conceptual framework*.* Source: Adapted and adjusted from literature review (2023).

Other determinants of data use in decision-making such as organizational (governance, planning, training, supervision, quality, promotion of culture of information, availability of resources), technical [complexity of reporting forms, health information system (HIS) design, computer software], and behavioral (level of knowledge of contents of EMRs, data quality checking skills, problem solving for HIS tasks, competence in HIS tasks, confidence) factors were adopted as per the original PRISM model (Figure 1).

The PRISM framework was adapted because it has been used by other scholars to study EMR system data use in Ethiopia (2) and Malawi (1).

## 2. Methods

### 2.1. Study design and setting

This is a facility-based cross-sectional analytical study design that uses a quantitative approach. This study was supplemented by document review to ascertain the existing EMR system data use practices in decision-making at the facility level. It was conducted in Dodoma Region, which is among the regions with an increasing trend in coverage of facilities utilizing EMR systems (18). Dodoma Region is one of the 31 administrative regions of Tanzania and it is located in Central Tanzania. It has a total of eight district councils. The region also has a total of 497 health facilities, including 26 hospitals, 69 health centers, and 402 dispensaries (23). Among the health facilities, 35 public primary health facilities have installed and use EMR systems, of which seven are district hospitals, 24 are health centers, and four are dispensaries (24).

### 2.2. Study participants

The study participants of this study were health facility managers randomly selected across 30 public primary health facilities with functional EMR systems. A sample size of 315 was estimated with an assumption of a 95% confidence interval, a 5% significance level, and a standard normal deviation of 1.96. The proportion of EMR utilization was set at 26.6% (2). A total of 308 health managers were eventually included in the data collection process. The eligibility criteria for participation were as follows: (1) position: health managers working in public primary health facilities with functional EMR systems, and (2) age: 18 years or older. The exclusion criteria included (1) not consenting to participate in the study, (2) using program-specific EMR systems such as CTC2 database, and (3) being on leave during the period of data collection.

A stratified sampling technique with proportionate allocation to each facility was employed. To recruit 315 participants from the selected facilities, Neyman's allocation formula as cited by Mathew (25) was used:

$$n_{h}\;=\;(N_{h}/N)\times n$$

where *n_h_* is the sample size for stratum *h*, *n* is the total sample size, *N_h_* is the population size per facility, and *N* is the total population.

**Figure 2:**
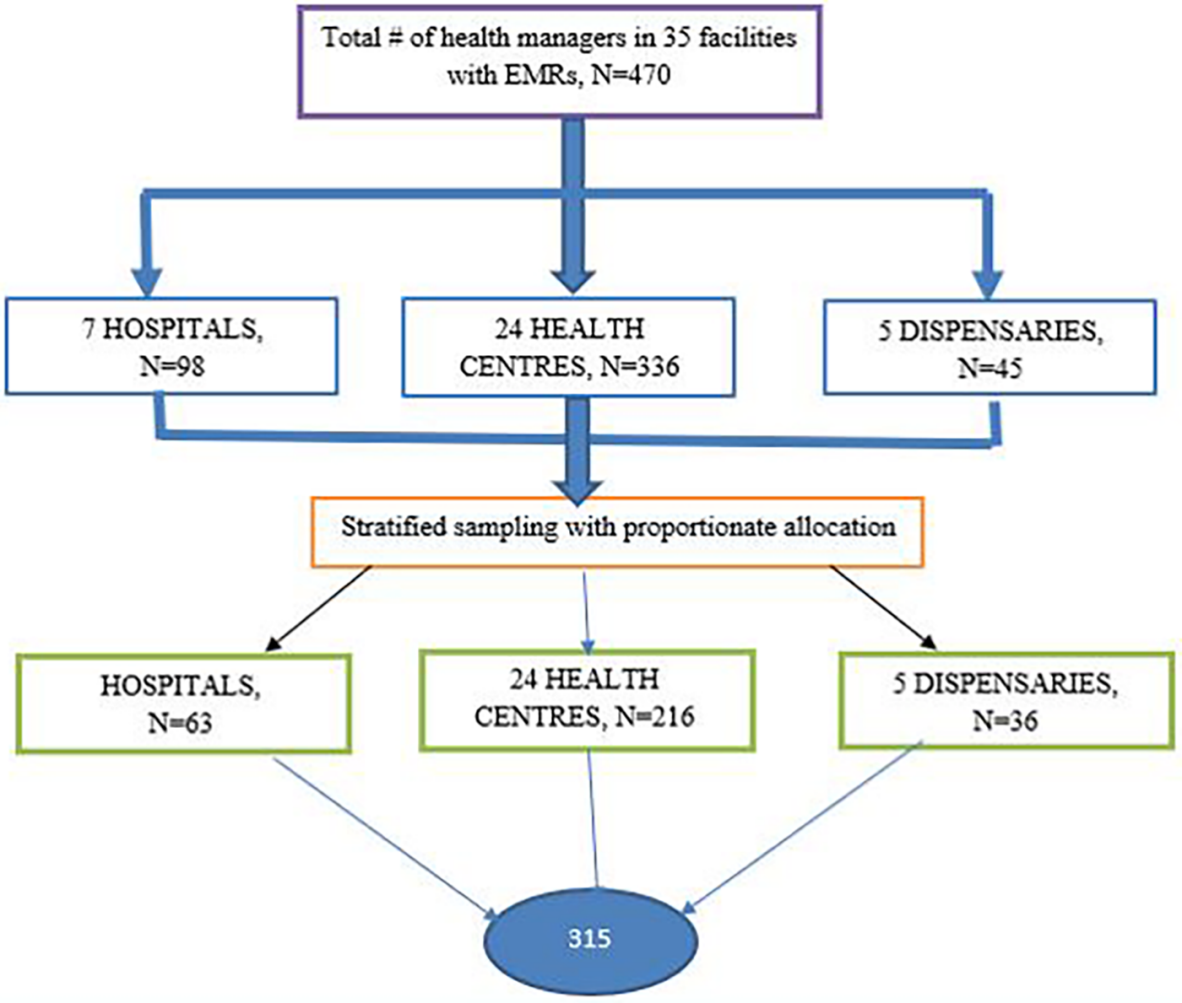
Schematic presentation of the sampling procedure for EMR system data use, Dodoma Region.

Samples were distributed according to their respective strata (hospital, health centre, or dispensary). The sample size per facility, *N_h_*, was determined by using the recommended number of HMT members, which for the hospital and health center is 14 members each and for the dispensary is nine members (26). Hence, the population of participants was sampled as shown (Figure 2).

### 2.3. Data collection methods

A structured self-administered questionnaire supplemented with documentary review was used to collect data on sociodemographic, organizational, behavioral, and technical factors as well as knowledge and attitudes regarding the use of EMRs. The tool was adapted from and constructed using PRISM (27) and was based on previous studies (2). The questionnaire was administered in English. Before administration, the questionnaire was designed using Kobo toolbox software, and diploma holder nurses were involved in administering the tool after 2 days of training.

A document review checklist was used to assess the existing EMR use in decision-making at the facility level over the past 6 months. The documents reviewed included HMT meeting minutes, commodity audit reports, commodity procurement reports, and annual facility planning reports. To ensure good data quality and avoid problems of missing data, the recruitment team were trained for 2 days. The respondents who were absent during data collection were repeatedly visited to minimize the rate of high non-responses.

### 2.4. Variable measurements

#### 2.4.1. Knowledge of computer and data use

This was assessed by using a 10 question score as follows: internet browsing, making calculations, sending email communication, managing the EMR database, checking accuracy of data, plotting data by months or years, computing trends from bar charts, explaining findings and their implications, using data for identifying gaps and setting targets, using data for making various decisions, and providing feedback. A mean score above or equal to 90 denoted excellent, 80–<90, very good, 60–<80, good, 50–<60, fair, and less than or equal to 50, poor (2).

#### 2.4.2. Attitude

This was assessed by using eight questions that could be answered on a scale (strongly disagree, disagree, neutral, agree, strongly agree). Those who scored an aggregate of above 32 indicated a positive attitude (good), and those who scored below 32 were considered as having a negative attitude (bad) (2).

#### 2.4.3. Perceived EMR system quality

This was assessed by using five questions that could be answered on a scale (strongly disagree, disagree, neutral, agree, strongly agree). Those who scored an aggregate score of above or equal to 20 indicated a positively perceived EMR system quality (good), and those who scored below 20 were considered as perceiving a negative EMR system quality (poor) (2).

#### 2.4.4. Perceived EMR service quality

This was assessed by using nine questions that could be answered on a scale (strongly disagree, disagree, neutral, agree, strongly agree). Those who scored an aggregate score of above or equal to 36 indicated a positively perceived EMR service quality (good) and those who scored below 36 were considered as perceiving a negative EMR system service quality (poor) (2).

#### 2.4.5. Perceived EMR information quality

This was assessed by using seven questions that could be answered on a scale (strongly disagree, disagree, neutral, agree, strongly agree). Those who scored an aggregate score of above or equal to 28 indicated a positively perceived EMR information quality (good) and those who scored below 28 were considered as perceiving a negative EMR system information quality (bad) (2).

#### 2.4.6. EMR use in decision-making (health manager)

This was defined as having ever used EMR system data in decision-making, and the extent of use was assessed by a question with 10 items on the participants use of the EMR to perform one or more of the following clinical and administrative/managerial functions: (1) support quality improvement (QIT), (2) assess adherence to clinical practice guidelines, (3) create a dashboard with measures of organizational performance (e.g., revenue collection), (4) identify high risk patients, (5) create individual provider performance profiles, (6) create dashboards with unit performance (e.g., trend in revenue collection), (7) generate reports to inform strategic planning, (8) identify care gaps for patients, (9) assess adherence to guidelines, (10) create an approach for clinicians to query for data (10). A mean score above 5 denoted adequate data use, and a mean score below 5denoted inadequate data use.

### 2.5. Data analysis

Data analysis was conducted by using the statistical software SPSS version 25. All filled out questionnaires were carefully reviewed by the data collectors for clarity and completeness. Data were coded, and data cleaning was performed before entry into the SPSS software. For this study, we conducted two main analyses corresponding to our objectives. The first was a descriptive analysis, to describe the proportion of study participants and the proportion of facilities using EMR data in decision-making. The aim of the second analysis was to determine the associations between technical, behavioral, organizational, technical, and demographic factors and EMR data use in decision-making by using bivariable and multivariable logistic regression analyses. All variables were subjected to chi-square test and then predictor variables with outcome at a *p*-value below 0.2 were considered for the logistic regression analyses. All statistics with a *P*-value < 0.05 were regarded as significant.

## 3. Results

### 3.1. Sociodemographic characteristics of study participants

**Figure 3:**
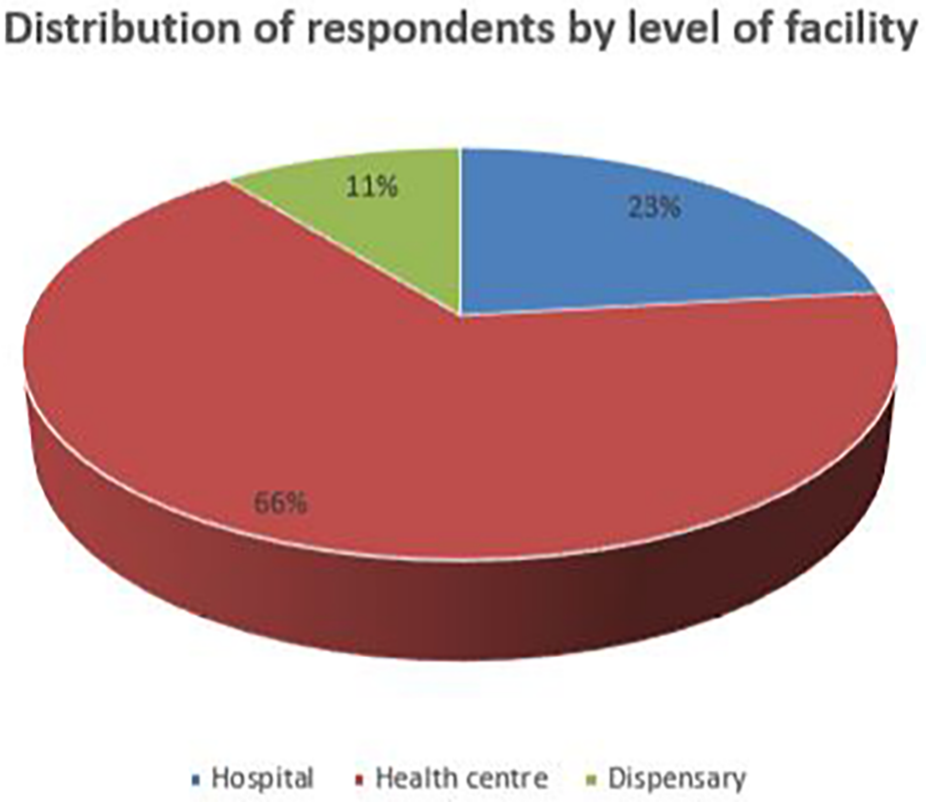
Distribution of respondents by level of facility.

A total of 308 health managers from different categories and educational levels out of 315 sampled participants with a mean age of 33 years were interviewed. The majority (53.9%) of the respondents were men. Among the study participants, 118 (38.3%) were aged less than 30 years, 141 (45.8%) were aged 30–40 years, and 49 (15.9%) were aged 40 years and above. In addition, 75 (24%) had attended undergraduate degrees. Approximately 39.9% of the study participants had work experience of more than 6 years. In addition, most of the respondents (42.9%) were nurses compared with other professions (Table 1). However, the majority 204 (66.2%) of the facility health managers were from health centers, and only 33 (11%) were from dispensaries (Figure 3).

**Table 1:** Distribution of social demographic characteristics of study respondents (*n* = 308).

Variable	Category	Frequency	Percentages (%)
Study site	Dodoma CC	39	12.7
	Kongwa DC	55	17.9
	Chamwino DC	84	27.3
	Kondoa TC	44	14.3
	Kondoa DC	28	9.1
	Bahi DC	12	3.9
	Chemba DC	46	14.9
Residence	Urban	146	47.4
	Rural	162	52.6
Sex	Male	166	53.9
	Female	142	46.1
Age (years)	≤29	118	38.3
	30–40	141	45.8
	≥40	49	15.9
Education	Certificate	64	20.8
	Diploma	169	54.9
	Degree and above	75	24.4
Profession	General practitioners	70	22.7
	Nurse/midwives	132	42.9
	Medical laboratory	26	8.4
	Pharmacists	28	9.1
	Others*	52	16.9
Managerial position	Facility incharge	31	10.1
	Matron/patron	31	10.1
	Pharmacy incharge	31	10.1
	Laboratory incharge	30	9.7
	Facility accountant	19	6.2
	Health secretary	5	1.6
	OPD incharge	31	10.1
	HMIS focal person	17	5.5
	Others*	113	36.6
Work experience	≤6 years	185	60.1
	>6 years	123	39.9
Administrative experience	≤1 year	92	29.9
	>I year	216	70.1

* Other cadres (laboratory staff, radiology staff, accountants, health secretary).

### 3.2. EMR system data use practices in decision-making among health managers

EMR use in decision-making among health managers was measured if a participant reported use of the EMR data in decision-making and clarified the purpose of EMR data use. The majority of the participants, 174 (56.5%), reported that they currently used EMR in their facility, and 128 (73.6%) used it on a daily basis. Approximately 142 (46.1%) of the participants had used the EMR system before. Among the respondents, approximately 125 (40.6%) reported the use of EMR system data in decision-making, and among them, approximately 103 (33.4%) had adequate EMR data use practices (Table 2).

**Table 2:** EMR system data use practices among health managers in public primary health facilities, Dodoma Region (*n* = 308).

Variable		Frequency	Percent
Past EMR use	No	166	53.90
	Yes	142	46.10
Current EMR use	No	134	43.51
	Yes	174	56.49
Frequency of EMR use	Daily/all the time	128	73.56
	Three times a week	35	20.11
	Once a week	3	1.72
	I do not remember exactly	8	4.60
EMR data use	No	183	59.42
EMR data use	Yes	125	40.58
Extent of data use	Inadequate data use	205	66.56
	Adequate data use	103	33.44

**Figure 4:**
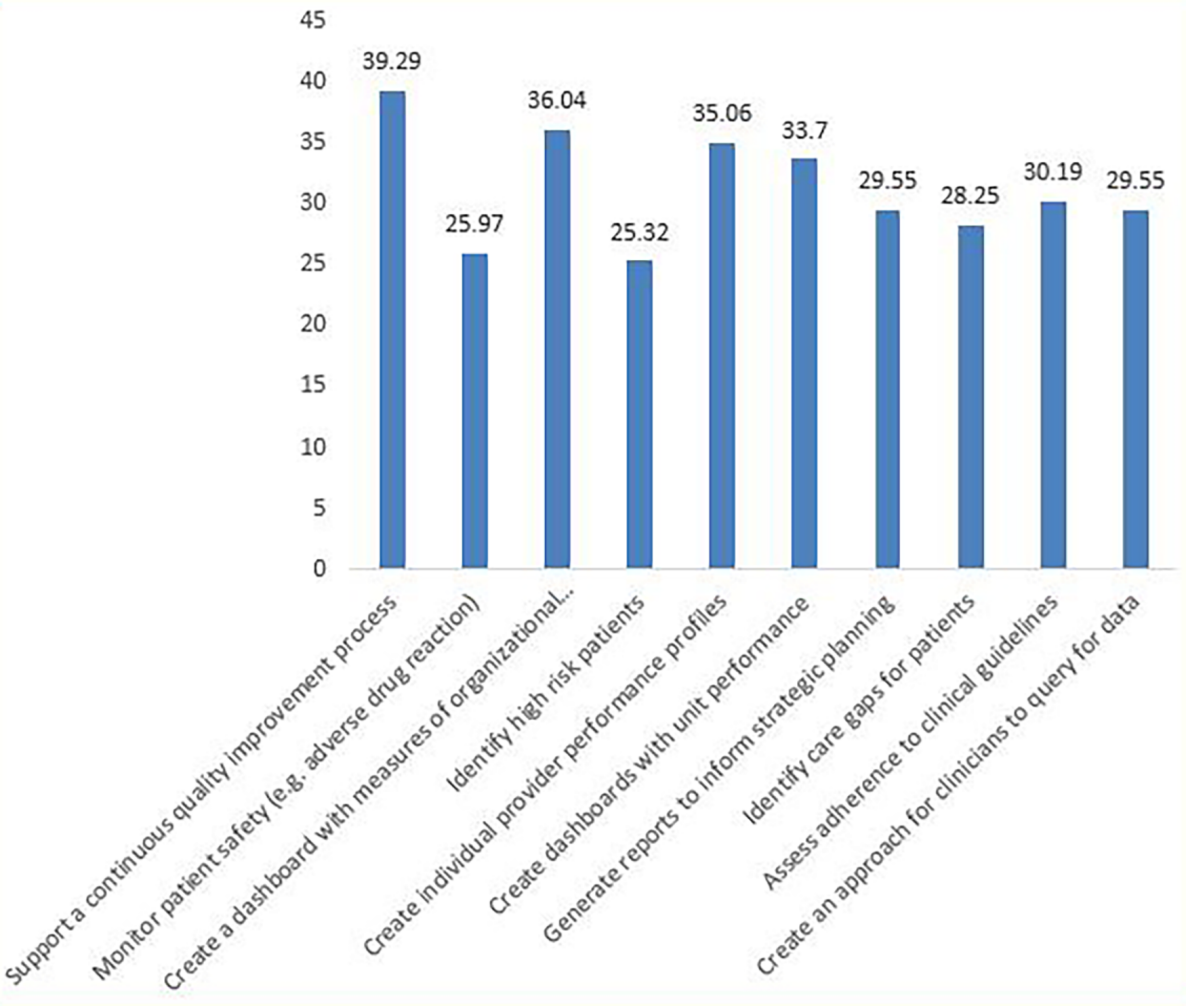
Purpose of EMR use by health facility managers.

The EMR data were most commonly used for continuous quality improvement (121,39.29%) and were least commonly used for identifying high-risk patients (78, 25.32%) (Figure 4).

**Figure 5:**
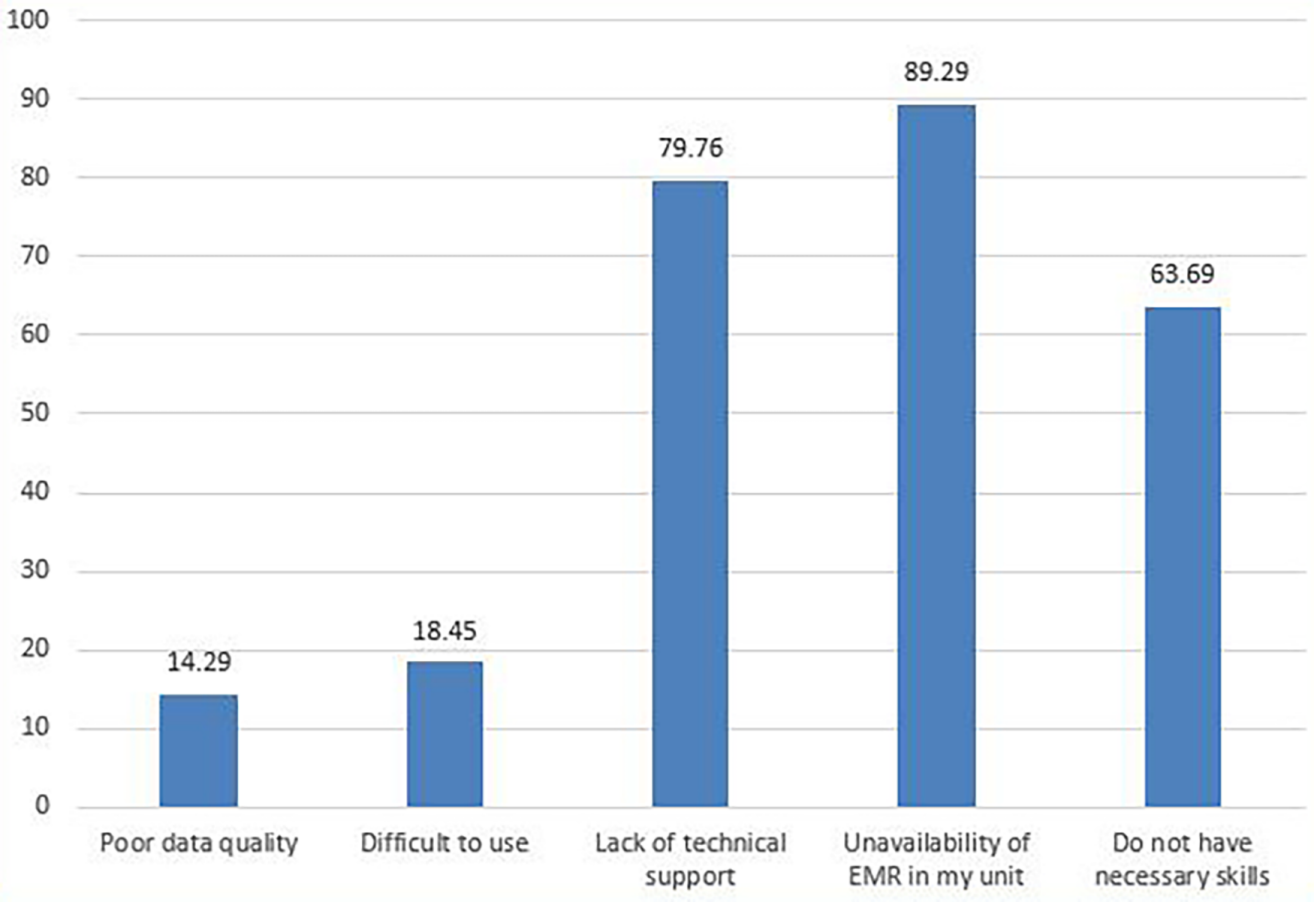
Reasons for not using EMR data in decision-making frequently (%).

Furthermore, approximately 168 (54%) of the managers did not use EMR in decision-making frequently, and the main reported reason for not using the system frequently in decision-making was the unavailability of the EMR system in some units/departments (150, 89.3%) (Figure 5).

#### 3.2.1. EMR system data use and related organizational characteristics

Overall, 184 (59.7%) of the participants had access to at least one computer, and among them, 19 (10.4%) shared the computer with more than four people. Furthermore, 120 (39%) of the participants had been trained on HMIS, while 152 (49.4%) had been trained on EMR use. However, 174 (56.5%) of the health managers are currently using the EMR system.

Most of the participants, 263 (85.4%), held regular discussions on EMR during facility meetings, and 180 (60.0%) reported that there was facility management support in the use of EMR system data. Approximately 109 (35.4%) of the participants mentioned that there was a person assigned to the facility to facilitate EMR use within the institution as an EMR champion (Table 3).

**Table 3:** Organizational factors and EMR system data use, Dodoma Region (*n* = 308).

Variable	Response	Frequency	Percentages (%)
Availability of computer	Yes	184	59.7
	No	124	40.3
Number of accessible computers	1	132	71.7
	2	25	13.6
	3	8	4.3
	4 and above	19	10.4
Functionality of computer	Yes	165	89.7
	No	19	10.3
Sharing computers	Yes	124	67.4
	No	60	32.6
Training on EMR	Yes	152	49.4
	No	156	50.6
Discussion on EMR	Yes	263	85.4
	No	45	14.6
Motivation on EMR use	Yes	180	60.0
	No	120	40.0
Trained on HMIS	Yes	120	39.0
	No	188	61.0
Current use of EMR system	Yes	174	56.5
	No	134	43.5
Availability of EMR champion	Yes	109	35.4
	No	199	64.6

#### 3.2.2. EMR system data use in decision-making and related behavioral characteristics

Preference of the EMR system compared with the paper-based system, attitude, and knowledge of computer and data use were assessed. Among the participants, 288 (93.51%) reported that they prefer the EMR system over the paper-based system. Knowledge of computer and data use ranged from 67.53% to 93.83% in all 10 areas assessed. Furthermore, 267 (86.69%) of the health managers had a good attitude toward the EMR system (Table 4).

**Table 4:** Behavioral factors and EMR system data use, Dodoma Region (*n* = 308).

Variables	Response	Frequency	Percentages (%)
Prefer EMR than paper-based system	Yes	288	93.51
	No	20	6.49
Attitude level	Poor	41	13.31
	Good	267	86.69
Knowledge of computer and data use			
1. Internet browsing	Yes	289	93.83
	No	19	6.17
2. Calculations	Yes	275	89.29
	No	33	10.71
3. Email	Yes	285	92.23
	No	23	7.47
4. EMR database management	Yes	157	50.97
	No	151	49.03
5. I can check data accuracy	Yes	223	72.4
	No	85	27.6
6. I can plot data by months or years	Yes	239	77.6
	No	69	22.4
7. I can compute trends from bar charts	Yes	272	88.31
	No	36	11.69
8. I can explain findings and their implications	Yes	219	71.1
	No	89	28.9
9. I can use data for identifying gaps and setting targets	Yes	209	67.86
	No	99	32.14
10. I can use data for making various types of decisions and providing feedback	Yes	208	67.53
	No	100	32.47

#### 3.2.3. EMR system data use and related technical characteristics

Almost all the participants (289, 93.8%) reported that they were able to use a computer. A total of 134 (43.35%) had positively perceived EMR system quality but only 22 (7.14%) had positively perceived EMR system service quality (Table 5).

**Table 5:** Patterns of technical factors and EMR use among health managers, Dodoma Region (*n* = 308).

Variables	Response	Frequency	Percentages (%)
Ability to use computer	Yes	289	93.83
	No	19	6.17
Perceived EMR system quality	Poor	174	56.49
	Good	134	43.51
Perceived EMR service quality	Poor	286	92.86
	Good	22	7.14
Perceived EMR information quality	Poor	123	39.94
	Good	185	60.06

### 3.3. Factors associated with EMR system data use in decision-making

Binary logistic regression analysis was used to determine how the independent variables influenced the dependent variable collectively. The analysis was also meant to establish the extent to which each independent variable affected the dependent variable and which factors were more significant.

The following factors were found to independently influence EMR data use: age of respondent, education level, access to a computer, motivation to use data, functionality of the computer, perceived EMR system information quality, and computer and data use skills as provided in Table 6.

**Table 6:** Factors associated with EMR data use among health managers, Dodoma Region (*n* = 308).

Variable	Response	EMR data use		COR (95% CI)	AOR (95% CI)	*P*-value
		Adequate data use, *n* (%)	Inadequate data use, *n* (%)			
Level of education	Certificate	8 (7.77)	56 (27.32)	1	1	
	Diploma	58 (56.31)	111 (54.15)	3.66 (1.63, 8.18)	3.24 (1.29, 8.15)	0.012
	Degree or above	37 (35.92)	38 (18.54)	6.82 (2.86, 16.23)	5.26 (1.84, 7.15)	0.002
Cadre	Nurses	29 (28.16)	103 (50.24)	1	1	
	Specialists/general practitioners	35 (17.07)	35 (33.98)	3.55 (1.90, 6.63)	2.64 (1.17, 5.94)	0.019
	Other cadres*	39 (37.86)	67 (32.68)	2.07 (1.17, 3.66)	1.53 (0.76, 3.08)	0.236
Managerial experience	>1	83 (80.58)	133 (64.88)	1	1	
	≤1	20 (19.42)	72 (35.12)	0.45 (0.25, 0.78)	0.38 (0.19, 0.74)	0.004
Have access to computer	No	12 (111.65)	112 (54.63)	1	1	
	Yes	91 (88.35)	93 (45.37)	9.13 (4.71, 17.07)	4.72 (1.65, 13.48)	0.004
Discussion on EMR in meetings	No	7 (6.80)	38 (18.54)	1	1	
	Yes	96 (93.20)	167 (81.46)	3.12 (1.34, 7.26)	2.77 (1.01, 7.58)	0.047
Degree of EMR implementation	Partial	75 (72.82)	193 (94.15)	1	1	
	Fully	28 (27.18)	12 (5.85)	6.00 (2.90, 12.42)	7.23 (3.15, 16.59)	0.001
Perceived EMR information quality	Poor	26 (25.24)	97 (47.32)	1	1	
	Good	77 (74.76)	108 (52.68)	2.66 (1.56, 4.48)	2.84 (1.50, 5.39)	0.001
Ability to use computer	No	6 (5.83)	13 (6.34)	1	1	
	Yes	97 (94.17)	192 (93.66)	1.09 (0.40, 2.97)	0.12 (0.019, 0.79)	0.028
Knowledge of computer and data use	Poor	6 (5.83)	36 (17.56)	1	1	
	Fair	1 (0.97)	15 (7.32)	0.40 (0.044, 3.61)	1.017 (0.085, 1.21)	0.989
	Good	6 (5.83)	52 (25.37)	0.69 (0.21, 2.31)	2.35 (0.36, 1.54)	0.372
	Very good	8 (7.77)	12 (5.85)	4.1 (1.15, 1.38)	1.3 (2.02, 9.14)	0.007
	Excellent	82 (79.61)	90 (43.9)	5.46 (2.19, 3.45)	1.84 (3.38, 10.13)	0.001

* Other cadres (laboratory staff, radiology staff, accountants, health secretary).

#### 3.3.1. Sociodemographic factors

Health managers with a diploma and a degree or above were three and five times more likely to use the EMR system than certificate holders, respectively [AOR = 3.24 with 95% CI of (1.29, 8.15), *p* = 0.012 and AOR = 5.26 with 95% CI of (1.84, 7.15), *p* = 0.002]. In addition, specialists/general practitioners were 2.64-fold more likely to use EMR system data than other cadres [AOR = 2.64 with 95% CI of (1.17, 5.94), *p* = 0.019]. Those who were working in rural areas were less likely to use EMR data than those working in urban areas [AOR = 0.46 with 95% CI of (0.26, 0.81), *p* = 0.008]. Those having managerial experience of ≤1 year were less likely to be associated with EMR system data use [AOR = 0.38 with 95% CI of (0.19, 0.74), *p* = 0.004]. However, other factors, such as age, sex, and type of facility did not have significant findings.

#### 3.3.2. Organizational factors

Those who reported having access to computers and discussions on EMR during meetings were 4.72 and 2.77 more likely to use EMR system data adequately compared with others, respectively [AOR = 4.72 with 95% CI of (1.65, 13.48), *p* = 0.004 and AOR = 2.77, 95% CI: 1.01, 7.58, *p* = 0.047]. Those who reported having EMR systems in all working areas were seven times more likely to use EMR system data in decision-making than those who reported having EMR systems in some units [AOR = 7.23 with 95% CI of (3.15, 16.59), *p* = 0.001]. Other factors did not have significant findings.

#### 3.3.3. Behavioral factors

Respondents with good perceived EMR system information quality were three times more likely to use EMR systems than those with poor perception of system quality [AOR = 2.84 with 95% CI of (1.50, 5.39), *p* = 0.001]. However, preference for the EMR system and attitude toward EMR did not have significant findings.

#### 3.3.4. Technical factors

Health managers who had excellent knowledge of computer and data use were two times more likely to use EMR system data than those with poor knowledge of computer and data use [AOR = 1.84 with 95% CI of (3.38, 10.13), *p* = 0.001]. Interestingly, those who reported being able to use computers were less likely to use EMR system data adequately [AOR = 0.12 with 95% CI of (0.019, 0.79), *p* = 0.028].

## 4. Discussion

This study set out to assess the EMR system data use in decision-making and its associated factors in public primary health facilities. We assessed variables related to (1) sociodemographic, (2) organizational, (3) technical, and (4) behavioral factors.

The findings of this study revealed that approximately half (56.5%) of health managers were current users of the EMR system, of whom 73.6% used the system on a daily basis. Among them, approximately 125 (40.6%) used EMR data in decision-making. This rate was higher than that in a study conducted in Ethiopia, which revealed that EMR system data were used by 26.6% of the healthcare workers (2). Most of the facilities were partially electronic and partially paper-based. This finding is the same as those revealed in studies conducted in Ethiopia and Malawi (1, 2, 28).

The relatively high use of EMR system data may be attributed to the fact that most of the users preferred EMRs over paper-based systems (93.5%). In addition, respondents also had good computer and data use skills ranging from 50.93% to 93.83%, which might have contributed to this level of implementation. This is supported by a study conducted in Dire Dawa (2) and Eastern Ethiopia (28).

Health professionals with excellent knowledge of computers and data use skills were more likely to use EMR systems than others. This finding is similar to that from a study conducted in Tanzania, which shows that the knowledge gap and skills among healthcare workers significantly influence EMR system use and the data management process (16, 29). In a study conducted in Ethiopia, it was revealed that staff with good data analysis skills were more likely to use health information system data than others (30). Moreover, a study conducted at Muhimbili National Hospital revealed that one of the challenges in the implementation of EMRs was inadequate skills of healthcare workers in the use of computers, which was because most of the old staff were aging and had inadequate basic skills in the use of computers, and the new staff turnover at the facility was high. All this resulted in poor quality of clinical note documentation. Refresher trainings and interfacility forums to share real practical experience were recommended (31).

In this study, the age of the health managers did not significantly impact their likelihood of using EMR data. This was different from a study conducted in Ethiopia that revealed that health professionals in the age group of 26–30 years were 1.61 times more likely to use the EMR system than those whose age was more than 40 years (32). However, another study in Ethiopia revealed that health professionals with a higher age, above 35 years, were more likely to use EMR systems than others (28). The different outcomes might be influenced by contextual factors such as the availability of training programs, the complexity of the EMR system, and the overall technological environment in the healthcare setting.

In this study, it was further revealed that motivation (managerial support) for the use of EMR system data was less likely to be associated with improved use of EMR data. This is different from a study conducted in Bahir Dar City, Ethiopia, which revealed that healthcare workers with managerial support were more willing to use EMR systems than others (33). However, another study (34) revealed that good governance (leadership, participatory monitoring, regular review of data) was associated with improved data use practices. In addition, performance-based financing was also found to have a role in promoting data use. In another study too, conducted at Mount Meru Hospital, EMR use was limited by low knowledge and skills of healthcare workers on information and communication technology (ICT) and inadequate management support (35). Therefore, the differences in findings could be due to variations in the healthcare settings, organizational cultures, levels of managerial support, and overall readiness of healthcare workers to adopt and utilize EMRs.

Those who were working in rural areas were less likely to use EMR data compared with those working in urban areas. This is congruent with a study conducted in the USA (10) in which hospital characteristics influenced use of EHR data, with the small, rural, state/local government run, and non-teaching hospitals having the lowest rates of EHR data use. This might be because EMR adoption rates in rural areas are significantly lower than in urban areas. This could be attributed to factors such as technology access, limited resources, and lower EMR adoption rates in rural settings.

Furthermore, respondents with good perceived EMR information quality were three times more likely to use EMR system data than those with poor perceived information quality. However, in this study, perceived system quality and service quality did not have a significant influence on EMR use. This is different from a study conducted in Ethiopia in which EMR system service quality and system quality were independent predictors of EMR use (2). Another study conducted in Gabon revealed that perceived EMR system quality and information quality were 1.7 times more likely to impact use of the system (36). This may be because data use is influenced by how users perceive the quality of the data produced.

The degree of EMR implementation was also a predictor of EMR use. Those who had EMR systems in all departments/units were more likely to use EMR systems than others. This is congruent with a study conducted in Ayder Hospital in Ethiopia in which 95% of the units were using the EMR system; hence, EMR use was higher (37). Another study conducted in Kenya revealed that EMR use was not adequate due to infrastructure challenges (38). In addition, another study revealed that the availability of health information system resources, such as EMR systems, organizational structure, and training, potentially influences data use (34). This may be because the availability of the EMR system in all departments increases the chance that in hospital discussions, all staff will be discussing data generated from the EMR system rather than a mixture of sources of data.

Training on EMR use had no influence on EMR system data use in this study. This was in line with a study conducted in Dire Dawa, Ethiopia (2). However, this finding is in contrast with findings from other studies conducted in different regions of Ethiopia (28), Malawi (1), and Kenya (38). It is well known that training has great potential to improve EMR use, as it can improve the attitude, knowledge, and skills of health managers. While training is a crucial component, its effectiveness in promoting EMR data use is intertwined with other factors, such as managerial support, cultural considerations, and resource availability. Hence, a comprehensive approach that addresses these factors is essential for maximizing the benefits of EMRs implementation.

A higher education level was also associated with EMR system data use, in which those with a diploma and a degree or above were 3 and 5 times, respectively, more likely to use EMR system data than those with a certificate. This is congruent with a study conducted in Ethiopia in which health professionals with an educational level of first degree and above were 1.92 times more likely to use the EMR system than diploma holders (32). This finding suggests that providing higher education and training opportunities to healthcare professionals could enhance their ability to engage with EMR systems and leverage the data they provide. This could lead to improved decision-making processes and patient care.

Overall, the study identified several factors that influenced the use of EMR system data among health managers. These factors included education level, professional role, managerial experience, access to computers, discussions about EMR, system implementation status, perceived system quality, and computer and data use skills.

## 5. Conclusions

The electronic medical records system has a proven advantage of enabling easy retrieval of data, including data analysis and use. This can potentially lead to more informed decision-making in the health sector. However, in this study, almost half of the respondents were current users of the EMR system, and 40.6% of the health managers were using EMR data in decision-making. This indicates that although many health managers are using EMR systems, a notable portion of decision makers have not fully integrated EMR data into their decision-making practices. Thus, for improvement in EMR system data use, future investments in issues such as training, installation of the system in all units/departments, education, and integration of EMR data into established decision-making workflows should be advocated. Since it was found that training in itself is insufficient to improve use of EMR, other organizational aspects of work routine that may serve as a challenge must be considered. Many health projects focus on training and technical aspects rather than improving organizational culture. Therefore, a comprehensive approach that addresses these factors is essential for maximizing EMR use in decision-making.

## Data Availability

The raw data supporting the conclusions of this article will be made available by the authors, without undue reservation.
